# Erythroid-Specific Transcriptional Changes in PBMCs from Pulmonary Hypertension Patients

**DOI:** 10.1371/journal.pone.0034951

**Published:** 2012-04-24

**Authors:** Chris Cheadle, Alan E. Berger, Stephen C. Mathai, Dmitry N. Grigoryev, Tonya N. Watkins, Yumiko Sugawara, Sangjucta Barkataki, Jinshui Fan, Meher Boorgula, Laura Hummers, Ari L. Zaiman, Reda Girgis, Michael A. McDevitt, Roger A. Johns, Frederick Wigley, Kathleen C. Barnes, Paul M. Hassoun

**Affiliations:** 1 Division of Allergy and Clinical Immunology, Johns Hopkins University School of Medicine, Baltimore, Maryland, United States of America; 2 Division of Pulmonary/Critical Care Medicine, Johns Hopkins University School of Medicine, Baltimore, Maryland, United States of America; 3 Medical Genetic Core, Children's Mercy Hospitals and Clinics, Kansas City, Missouri, United States of America; 4 Division of Rheumatology, Johns Hopkins University School of Medicine, Baltimore, Maryland, United States of America; 5 Division of Hematology, Johns Hopkins University School of Medicine, Baltimore, Maryland, United States of America; 6 Department of Anesthesiology and Critical Care Medicine, Johns Hopkins University School of Medicine, Baltimore, Maryland, United States of America; Agency for Science, Technology and Research – Singapore Immunology Network, Singapore

## Abstract

**Background:**

Gene expression profiling of peripheral blood mononuclear cells (PBMCs) is a powerful tool for the identification of surrogate markers involved in disease processes. The hypothesis tested in this study was that chronic exposure of PBMCs to a hypertensive environment in remodeled pulmonary vessels would be reflected by specific transcriptional changes in these cells.

**Methodology/Principal Findings:**

The transcript profiles of PBMCs from 30 idiopathic pulmonary arterial hypertension patients (IPAH), 19 patients with systemic sclerosis without pulmonary hypertension (SSc), 42 scleroderma-associated pulmonary arterial hypertensio patients (SSc-PAH), and 8 patients with SSc complicated by interstitial lung disease and pulmonary hypertension (SSc-PH-ILD) were compared to the gene expression profiles of PBMCs from 41 healthy individuals. Multiple gene expression signatures were identified which could distinguish various disease groups from controls. One of these signatures, specific for erythrocyte maturation, is enriched specifically in patients with PH. This association was validated in multiple published datasets. The erythropoiesis signature was strongly correlated with hemodynamic measures of increasing disease severity in IPAH patients. No significant correlation of the same type was noted for SSc-PAH patients, this despite a clear signature enrichment within this group overall. These findings suggest an association of the erythropoiesis signature in PBMCs from patients with PH with a variable presentation among different subtypes of disease.

**Conclusions/Significance:**

In PH, the expansion of immature red blood cell precursors may constitute a response to the increasingly hypoxic conditions prevalent in this syndrome. A correlation of this erythrocyte signature with more severe hypertension cases may provide an important biomarker of disease progression.

## Introduction

Pulmonary arterial hypertension (PAH) is a vascular disease that carries significant morbidity and mortality [1], [2], [3]. Morbidity and mortality rates vary and depend on the age, the degree of pulmonary hypertension, and the response to vasodilator therapy. Death as a result of both acute and chronic right heart failure may occur. PAH is currently characterized by uncontrolled cell proliferation and inflammation involving the pulmonary vascular resistive vessels, leading to a progressive increase in pulmonary vascular resistance, right ventricle hypertrophy, and eventual heart failure.

PAH can complicate connective tissue diseases such as scleroderma [4], [5] as well as other autoimmune diseases such as systemic lupus erythematosus and rheumatoid arthritis [6], [7]. Scleroderma, or systemic sclerosis (SSc), is a chronic multisystem autoimmune disease characterized by a vasculopathy, diffuse fibrosis of skin and various internal organs, and immune abnormalities. Up to 10–15% of SSc patients eventually develop PAH [8]. While the factors which lead a subset of SSc patients to go on to develop PAH are unclear, it is of great clinical interest to develop early markers of SSc-associated PAH (SSc-PAH) disease in order to provide more aggressive treatment to the “at risk” patient population. The early phase of the disease process has been difficult to study rigorously because of the delay in the diagnosis of SSc-PAH and the lack of reliable biomarkers for early disease. Lung biopsy is avoided since it is invasive and unsafe in these patients. For this reason we chose to use gene expression profiling of peripheral blood mononuclear cells (PBMCs) as a surrogate tissue which is readily obtainable from patients and provides a large pool of gene transcripts shown to have the potential to be highly sensitive to the disease microenvironments on a systems wide basis [9], [10]. Previous studies using PBMCs have demonstrated an ability to discriminate between PAH, in general, and healthy controls, identify PAH-specific genes [11], as well as distinguish between IPAH and SSc-PAH gene signatures [12]. However, more recent studies have shown considerable heterogeneity when examining directly the contrast in gene expression profiles in PBMCs from SSc-PAH and SSc patients [13], [14], [15]. For example, while Pendergrass et al. [13] focused on the increased expression of 9 genes that distinguished SSc-PAH from SSc, Risbano et al. [14] describe 5 genes which when down-regulated distinguish SSc-PAH from SSc patients. We hypothesized that chronic exposure of PBMCs to elevated pulmonary pressures in remodeled pulmonary vessels will be reflected by specific transcriptional changes in these cells and distinguish PH of various etiologies from both SSc and normal controls.

## Results

### Expression Profiling

**Figure 1:**
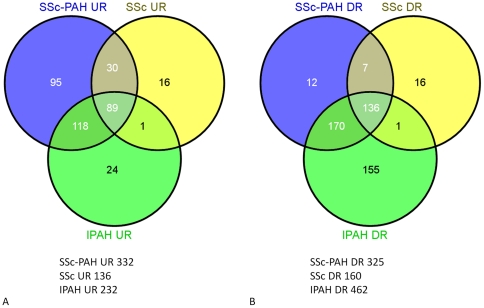
Venn diagrams illustrating the distribution of statistically significant disease-specific changes in gene expression. A) up-regulated and B) down-regulated gene expression for comparisons between SSc, SSc-PAH, and IPAH patients versus healthy controls. Total number of calculated differentially expressed genes for each comparison are as indicated below each diagram.

**Figure 2:**
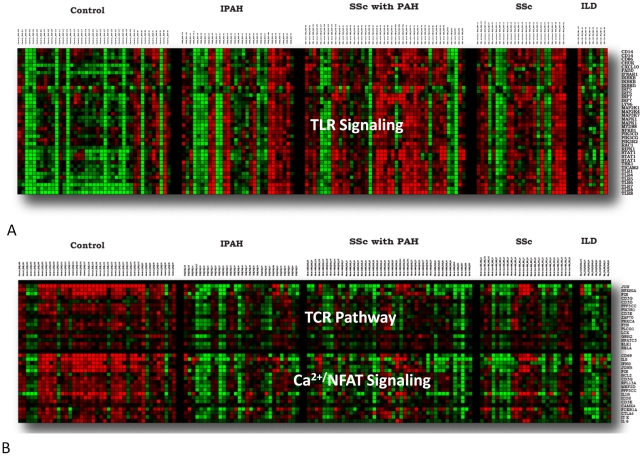
Heat map illustration of the distribution of gene expression among all samples for genes selected by pathways. A) TLR (Toll-like Receptor) Signaling, B) TCR (T cell receptor) Pathway, Ca^2+^/NFAT Signaling. Red indicates increase and green indicates decrease in relative gene expression for each gene calculated individually across all samples.

The transcript profiles of PBMCs from 42 SSc associated PAH (SSc-PAH) patients, 30 IPAH patients, 19 patients with SSc, and 8 patients with SSc complicated by interstitial lung disease and PH (SSc-PH-ILD) were compared to the transcript profiles of PBMCs from 41 healthy individuals (see Table 1 for a summary of demographic and clinical patient descriptions, see Table S3 for all relevant clinical data and descriptions, on a patient-by-patient basis). In our initial data analysis we compared the gene expression for each disease group versus that of the controls (as described in Methods) and found large numbers of significantly regulated genes between patients and controls (Figure 1). Among the 89 genes significantly up-regulated in common among IPAH and SSc with or without accompanying PH were components of the Toll-like receptor (TLR) signaling pathway including the STAT1, TLR7, and TLR8 genes. An expanded search of all genes in the TLR signaling pathway revealed a broad general trend (Figure 2A) of up-regulation of these genes in each disease group (ttest p-values of the average expression of these pathway genes by group versus controls were p = 0.04 for IPAH, p = 0.0006 for SSc-PAH, and p = 0.004 for SSc, SSc-PH-ILD was not significant at p = 0.09) altogether suggesting a chronic activation of the innate immune system in these patients. Scleroderma patients with or without PAH demonstrated a pattern of 30 distinct immune response genes (DAVID [NIH Database for Annotation, Visualization, and Integrated Discovery]: defense response; p<7×10^−4^) up-regulated in common including CXCL10, FCGR1A, HCK, MX2, NCF1C, PARP4, and TLR4 (see Table S1 for all cited genes, enrichment results, and complete group comparison results).

**Table 1:** Demographic and clinical characteristics of the patients with PH.

Variable	Overall (n = 140)	Control (n = 41)	SSc (n = 19)	IPAH (n = 30)	SSc-PAH (n = 42)	SSc-PH-ILD (n = 8)
Age (mean ± SD years)	53±13	45±12	55±10	52±12	60±13	61±11
Gender n (%)						
Female	117 (83.6)	34 (82.9)	19 (100)	25 (83.3)	33 (78.6)	6 (75.0)
Male	23 (16.4)	7 (17.0)	0 (0)	5 (16.7)	9 (21.4)	2 (25.0)
Race n (%)						
African American	20 (14.3)	7 (17.1)	0 (0)	5 (16.7)	8 (19.0)	0 (0)
Asian	4 (2.9)	1 (2.4)	0 (0)	1 (3.3)	1 (2.4)	1 (12.5)
Caucasian	115 (82.1)	33 (80.5)	19 (100)	23 (76.7)	33 (78.6)	7 (87.5)
Hispanic	1 (0.7)	0 (0)	0 (0)	1 (3.3)	0 (0)	0 (0)
NYHA functional class n (% of the PAH subjects)						
I	9 (11.3)	NA	NA	7 (23.3)	2 (4.8)	0 (0)
II	39 (48.7)	NA	NA	16 (53.3)	22 (52.4)	1 (12.5)
III	30 (37.5)	NA	NA	6 (20.0)	17 (40.4)	7 (87.5)
IV	2 (2.5)	NA	NA	1 (3.3)	1 (2.4)	0 (0)
NA (# for which value is not available)	60 (the non-PAH subjects)	41	19	0	0	0
6MWD (mean ± SD (# missing values))	1174±395 (64)	NA	NA	1383±392 (3)	1091±340 (1)	891±378 (0)
RA mean (mean ± SD (# missing values))	7.7±4.2 (61)	NA	NA	7.7±4.2 (0)	7.9±4.4 (1)	7.5±3.8 (0)
CI (mean ± SD (# missing values))	2.66±0.72 (61)	NA	NA	2.61±0.69 (0)	2.66±0.74 (1)	2.88±0.80 (0)
PVRI (mean ± SD (# missing values))	1062±573 (61)	NA	NA	1214±573 (0)	955±556 (1)	1042±610 (0)
PA saturation (mean ± SD (# missing values))	66.7±8.2 (65)	NA	NA	66.1±9.1 (2)	67.1±8.0 (2)	66.9±6.7 (1)

Except where indicated otherwise, values are the number (%).

Interestingly, PH patients (IPAH and SSc-PAH) shared a large number of distinct genes (118) (Figure 1A) that were significantly up-regulated in these PH groups but not in SSc including a total of seven genes CA2, HBA2, HBD, HBG1, HBG2, HBM, HBQ1 shown to be highly enriched for blood gas transport (DAVID: gas transport: p<7×10^−10^). This same group of 118 genes also contained a sub-group of genes involved in platelet biology (DAVID: platelet alpha granule: p<1×^−6^), a finding seen as well using MsigDB (Broad Institute Molecular Signatures Data Base) (platelet specific genes: p<1.6×^−12^).

The 136 genes down-regulated for all disease groups relative to healthy controls were significantly enriched for apoptosis (DAVID: apoptosis: p<1.4×^−4^), a trend that extended to the additional 170 down-regulated genes in common between SSc-PAH and IPAH (DAVID: regulation of apoptosis: p<3.0×^−6^). Further examination of down-regulated changes in gene expression individually in each condition using the methods of gene set analysis (webPAGE [16]) revealed a common trend of down-regulation of multiple pathways involved in T cell function (Table 2). Examination of several of these pathways in detail (Figure 2B) demonstrated a clear pattern of down-regulation of genes in these pathways across all patient groups relative to controls.

**Table 2:** Gene Set Analysis – selected pathway results illustrate a strong downward regulation of the adaptive immune response across multiple pathways for all disease groups versus healthy controls regardless of patient diagnosis.

GeneSet	Annotation	IPAH_v_Control	SSc-PH-ILD_v_Control	SSc-PAH_v_Control	SSc_v_Control
*TOB1 PATHWAY*	BioCarta	−4.620	−4.205	−5.096	−6.653
*CA^2+^NFAT SIGNALING*	GEArray	−5.695	−3.866	−5.047	−5.395
*IL12 PATHWAY*	BioCarta	−3.757	−3.740	−4.826	−6.919
*TCR PATHWAY*	BioCarta	−4.963	−4.673	−5.085	−4.813
*CCR5 PATHWAY*	BioCarta	−4.488	−3.189	−5.027	−5.916
*ARE/NRF2 PATHWAY*	BioCarta	−4.174	−2.190	−4.210	−5.027
*IL2PATHWAY*	BioCarta	−3.685	−3.575	−2.811	−3.572
*TH1-TH2-TH3 GENES*	SABiosciences	−3.263	−3.289	−1.938	−4.736

Enrichment scores are derived using the PAGE technique (see Methods) and are equivalent to (gene set size adjusted) Gaussian distributed z scores. The negative sign direction of the scores indicates that for each of the indicated pathways and comparisons the average gene expression is lower in the disease group than in controls. Shaded pathways (Ca2+NFAT signaling and the TCR Pathway) are broken out on a gene by gene and sample by sample basis in Figure 2.

### Signature Identification

**Figure 3:**
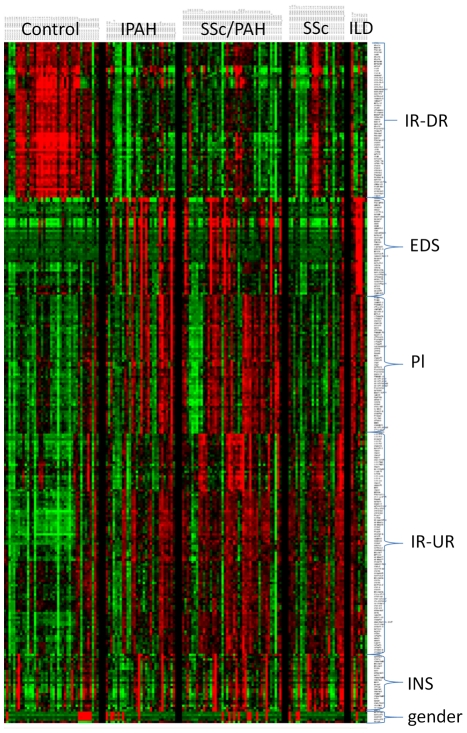
Heat map of unsupervised clustering (genes only) of 296 genes selected for both high variance (from a set of 500 most variant genes) and also as representative of six major patterns of distinctly correlated genes across the dataset. These patterns included an Immune Response signature (up- or down-regulated between controls and disease: IR-UR and IR-DR, respectively), the Erythroid Differentiation Signature (EDS) a Platelet specific signature (Pl), and an Immature Neutrophil Signature (INS). The gender specific cluster acts as a positive control, the specific signatures are discussed in detail in the text, particularly that of the EDS.

Unsupervised clustering of 296 genes (chosen from a pool of the 500 most variable genes and which displayed distinct clustering patterns) across all samples shows a clear trend of multiple clusters of correlated gene expression (Figure 3). Many of these clusters overlap the groups noted above. For example, in the first cluster, Immune Response genes down-regulated (IR-DR), 56 of the 58 genes depicted in this cluster are members of the group of the statistically down-regulated genes shared by all three major disease groups (IPAH, SSc-PAH, and SSc). Conversely, over half of the genes (41/81) in the Immune Response genes up-regulated (IR-UR) cluster are members of the group of the statistically up-regulated genes shared by all three major disease groups (IPAH, SSc-PAH, and SSc). Additional clusters included a gene expression signature originally identified in neutrophils [17] and found as an immature neutrophil signature (INS) in the PBMC fraction of blood from both vasculitis related to Wegener's granulomatosis [18] and systemic lupus erythematosus [19] patients relative to healthy controls, presumably as part of an aggravated chronic immune response. In this study the INS signature, when present, appears to be distributed nonspecifically among all samples. Although these three signatures (IR-UR, IR-DR, and INS) were clearly identified and annotated they were of less interest to this study either because of their general non-specificity (INS) or because of their inability to distinguish among separate disease groups (IR-UR, IR-DR). Two other signatures, however, both of which had been identified by functional annotation during our initial analysis (the gas transport and platelet specific genes), were represented by clusters shown here also to be related. The average expression levels for the Illumina probes in these two distinct groups have a Pearson correlation r = 0.6 across all subjects (see Table S2 for a complete listing of the genes in these two clusters). A distinctive feature of the gene expression of these two signatures (Erythroid Differentiation Signature – EDS; and Platelet derived – Pl) is that they are specific primarily to disease groups of patients with PH studied in this cohort (IPAH, SSc-PAH, SSc-PH-ILD).

### Signature Characterization

**Figure 4:**
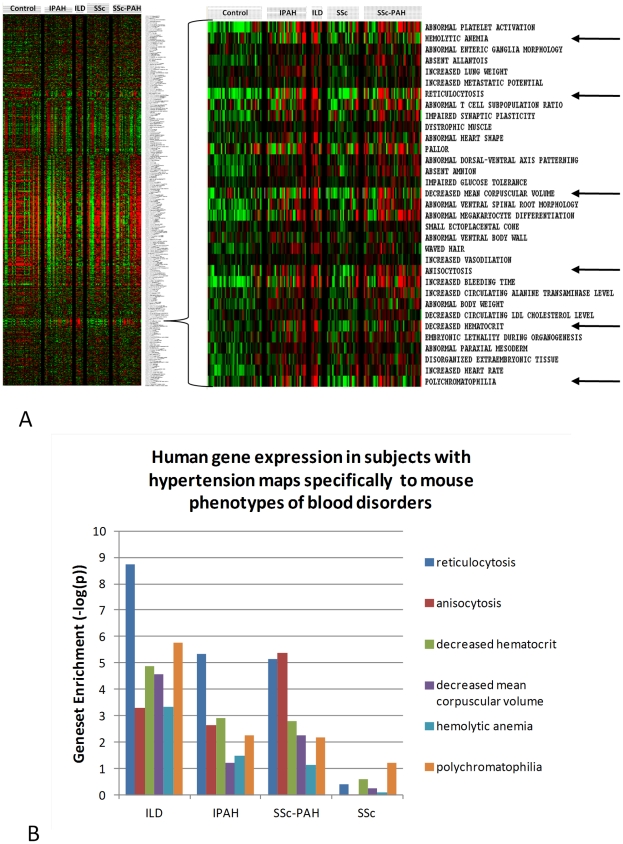
Results from gene set analysis of all gene expression data versus gene lists derived from the Mouse Genome Informatics (MGI) database. A) Gene set enrichment scores were calculated by the PAGE method using row normalized data for all (filtered) genes and samples. The scores were averaged by group and are normalized in the sense that that the average gene expression as scored by PAGE for any one gene set is expressed as the z transformation ((gene set size adjusted) of the average gene expression for that set. Disease specific patterns of gene set enrichment are as shown in the zoomed image. B) Average gene set expression in disease groups versus controls for various blood disorder-specific gene sets (as indicated by arrows in Fig. 4A) were tested by Students t test, the results being reported as the negative log (base 10) of the derived p-value (− log(p)).

In order to further characterize disease specific gene expression, all patient and control samples (see Methods) were directly evaluated by the methods of gene set analysis using gene lists derived from the Mouse Genome Informatics (MGI) database [20]. The file used for mouse phenotypic information contains 5011 distinct genes and 5142 phenotypic terms derived from information from specific gene mutations in multiple mouse strains, including an extensive library of specific gene knock-in and knock-out strains. In this analysis, patterns of gene expression among the PH, control and SSc groups were visualized across the entirety of the dataset by displaying the average gene expression for every mouse gene set for each human sample (Figure 4A). Patterns of disease specific gene set expression were detected which mapped to mouse gene lists involved in multiple phenotypes of blood disorders (Figure 4A, as indicated by arrows in the right panel). A summary of the differential expression p-value scores for these six blood disorder pathways when the individual disease groups were compared to controls shows a marked elevation of gene expression in the SSc-PH-ILD, IPAH, and SSc-PAH groups but not for SSc patients (Figure 4B). In particular, the contrast between SSc-PAH and SSc patients is striking.

The combination of elevated hemoglobin gene expression in patients with PH and the preliminary evidence that many differentially regulated genes in these disease groups mapped by gene set analysis to mouse blood disorder phenotypes suggested that human PH patients may be distinguished from healthy controls and SSc patients by a programmatic shift in gene expression in PBMCs, perhaps related to conditions of tissue hypoxia such as induced by PH. In order to further explore the functional implications of these regulated gene groups, a comprehensive list of genes was derived for the EDS and for the Pl groups by selecting all microarray probes in the complete dataset whose expression levels have a Pearson Correlation ≥0.7 with the average expression for the probes in each cluster (EDS; 42 Illumina probes, 40 unique genes, and Pl; 60 probes, 54 unique genes) as initially shown in Figure 3, across all the samples. This resulted in the identification of 169 Illumina probes representing 149 unique genes for the EDS, and 456 probes covering 384 unique genes for the Pl signatures, respectively (Table S2). These are the sets of probes and genes used for further analysis of these signatures.

### Tissue specificity of the EDS

**Figure 5:**
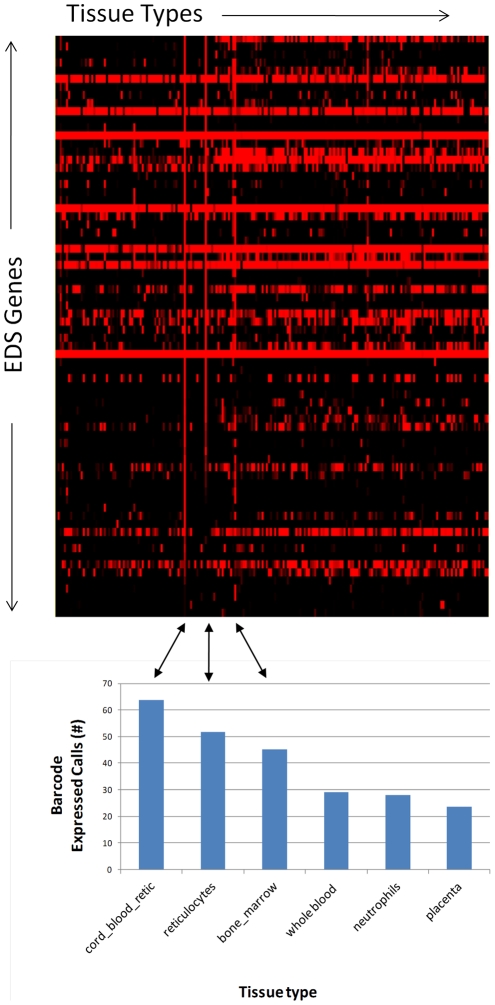
Gene Expression Barcode for EDS signature genes. The heat map is derived from individual barcode scores from zero (black) to one (red) with red indicating an increased likelihood that the gene is present in all samples in the experiment from which the tissue specific expression estimate is derived (see McCall et al. for complete description of the barcode algorithm [21]). As shown, the genes for the EDS signature were overrepresented for three tissues, in particular (cord blood reticulocytes, adult blood reticulocytes, and bone marrow), and their respective number of barcode expression calls are as indicated in the barchart directly below the corresponding areas of the barcode heatmap.

The full gene list for the EDS signature (149 unique genes) was next tested against the Gene Expression Barcode, a web-based tool [21] which allows for highly accurate estimates of tissue specific gene expression. Inspection of the EDS gene expression across a catalog of over 130 curated human tissue types derived from the GEO (Gene Expression Omnibus) and the ArrayExpress public repositories demonstrated that the expression of EDS genes as a group is highly restricted to a few distinctive cell types (Figure 5) of the hematopoietic lineage including a very high level of enrichment in reticulocytes and in bone marrow. Reticulocytes from cord blood were particularly enriched relative to reticulocytes from circulation. It is interesting to note in this context that cord blood has been well characterized as having a less mature reticulocyte population than adult blood [22].

**Figure 6:**
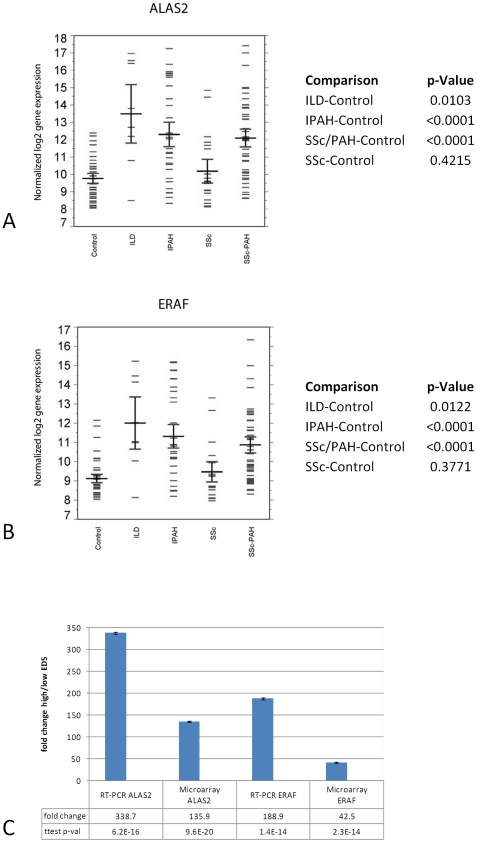
Statistically significant differential expression of erythroid CD71+ specific genes. A) ALAS2 and B) ERAF/AHSP genes in hypertension groups (SSc-PH-ILD, IPAH, and SSc-PAH) versus healthy controls and scleroderma (SSc). The box plots give the mean (horizontal black line), sample values (short blue lines), and 84% confidence interval (CI) (red lines) for each group, (non-overlap of the 84% CIs of two groups is an approximate indicator of significant difference between their means at the 0.05 level of significance). C) Individual ALAS2 and ERAF gene expression microarray results were validated by RT-PCR for high and low EDS patients across all hypertension classes (SSc-PAH, IPAH, SSc-PH-ILD). Further inspection of the EDS gene list also showed the inclusion of genes for both the GATA1 and KLF transcription factors which are both essential for erythroid development [28,29]. A test by gene set analysis of the entire dataset comparing each PH group directly versus the SSc group as the baseline group showed a significant and specific enrichment among genes which contain three different GATA transcription factor binding sites in their upstream promoter regions (TRANSFAC [30]) (Figure 7A). Interestingly, the genes up-regulated in each of the GATA transcription factor binding site gene sets were mostly non-overlapping either with each other (an average of 59–62% unique genes for each gene list) or with the EDS gene expression signature itself (95% unique non-overlapping genes) (Figure 7B) indicating that the effects of elevated GATA1 gene expression are both pervasive in the PH groups and are supplemental to the EDS gene expression signature itself. The observation that downstream regulatory events are related directly to EDS elevation (through the up-regulation of the GATA1 transcription factor gene) and are associated with PH groups, taken together with the previously demonstrated strong association of the EDS with reticulocyte maturation (Figure 5), led us to the identification of this signature as the Erythroid Development Signature (EDS).

Inspection of the EDS specific gene list showed markedly increased levels of expression for two genes in particular, ALAS2 (aminolevulinate, delta-, synthase2) (Illumina Probe ID [ILMN 2367126]) and ERAF (erythroid differentiation associated factor; recently renamed to AHSP [alpha hemoglobin stabilizing protein]) (Illumina Probe ID [ILMN 1696512]), (Figure 6 A&B) both essential for the terminal differentiation of erythroid cells [23]. The specific overexpression of ALAS2 and ERAF in hypertension samples was confirmed by RT-PCR (Figure 6C). The expression levels of these two genes track so well with the EDS gene expression signature overall (Pearson's correlation coefficient of r = 0.92 for ERAF and r = 0.95 for ALAS2 versus the average EDS gene expression across all samples) that we are using them as single gene candidate biomarkers to track EDS-specific gene expression elevation in patients currently enrolled in ongoing longitudinal studies. The tissue specific expression of both of these genes is restricted, primarily to fetal liver, bone marrow (in adults), and most abundantly in CD71+ early erythroid cells in the circulation (BioGPS http://biogps.gnf.org). We note that hemoglobin production in erythroid cells is one of the most important stages in their terminal differentiation [24], the induction of ALAS2 is essential for hemoglobin production [25] and ERAF/AHSP is essential for proper assembly of nascent alpha globin incorporation into hemoglobin-A [26]. In addition, conditions of hypoxia have been shown to directly result in elevated levels of ALAS2 in a human model of erythroid terminal differentiation [27].

**Figure 7:**
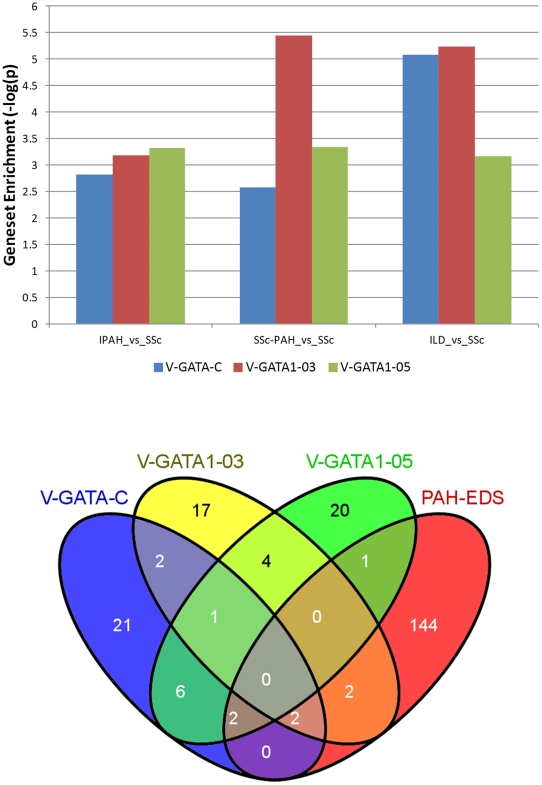
Results from gene set analysis using gene lists derived from the TransFac (gene associated transcription factor binding sites) database. A). The average changes in gene expression for patients with SSc-PH-ILD, IPAH, and SSc-PAH were tested versus patients with SSc. The p-values associated with the gene set enrichment scores are reported here as −log(p). B). Venn diagram illustrating overlap between the average up-regulated gene expression associated with the presence of 3 distinct GATA transcription factor binding sites. The EDS gene signature is included for comparison.

### External Validation of the Association of the EDS with Pulmonary Hypertension

In order to examine the extent of EDS association with PH, in general, beyond this current study, a survey was carried out of other published PH microarray studies (with and without SSc) in which PBMCs were the target tissue and for which full datasets were available through GEO (NCBI – Gene Expression Omnibus) [31]. A total of four datasets were identified, three of them recently published (in 2010), each of which contained at least one phenotype for PH (Table 3). Two of these datasets also included phenotypes for SSc both with and without accompanying PH (Risbano et al. [14] and Pendergrass et al. [13]). Each dataset was queried using the methods of gene set analysis using the EDS gene expression signature gene list embedded in a background of over 550 pathway gene lists derived from multiple sources. The strongest overall enrichment scores for the EDS were, not surprisingly, generated from the PH phenotypes of our own study (JHU; SSc-PH-ILD-33.3, IPAH-21.1, and SSc-PAH-17.9). The slightly positive enrichment score for the JHU SSc group reflects several patients who are EDS positive (and who may be progressing towards PH).

**Table 3:** Results from gene set analysis using the EDS as the query gene list. Enrichment scores are as described above.

Dataset	GEO #	SSc-PH-ILD	IPAH	SSc-PAH	SSc	PAH	IPF-PH	CBvAB
JHU	GSE33463	33.327	21.082	17.886	2.688	-	-	-
Risbano et al.^14^	GSE22356	-	26.019	7.553	−15.476	-	-	-
Pendergrass et al.^13^	GSE19617	-	-	15.004	9.247	-	-	-
Bull et al.^11^	GSE703	-	7.736	-	-	-	-	-
Rajkumar et al.^31^	GSE15197	-	-	-	-	4.083	−1.337	-
Goh et al.^32^	GSE6236	-	-	-	-	-	-	7.811

In all cases data was downloaded from the NCBI Gene Expression Omnibus (GEO) website as processed by the submitter and published by GEO as a Series Matrix File. Phenotype labels: Interstitial Lung Disease (SSC-PH-ILD), Idiopathic Arterial Hypertension (IPAH), Scleroderma with Pulmonary Arterial Hypertension (SSc-PAH), Scleroderma (SSc), Pulmonary Arterial Hypertension only (PAH), Idiopathic Pulmonary Fibrosis with Pulmonary Hypertension (IPF-PH), and human Cord Blood versus human Adult Blood (CBvAB).

In every external study examined, the PH phenotypes were significantly enriched for the EDS signature relative to controls and other non-PH phenotypes. In many cases the EDS gene list was the top scorer above all other pathways and signatures. In Risbano et al. [14] IPAH and SSc-PAH had scores of 26 and 7.6, respectively, while the SSc phenotype showed a strong negative enrichment of -15.5. An explanation for this anomaly can be seen by a sample by sample analysis using the gene expression of both ALAS2 and ERAF/AHSP as surrogate EDS biomarkers (Figure S1). In the Risbano et al. study [14], for reasons unclear, the EDS is suppressed in SSc patients relative to controls, while both the IPAH and the SSc-PAH groups contain patients with elevated biomarker levels relative to either controls or SSc groups. Conversely, in Pendergrass et al [13], patients with a diagnosis of SSc-PAH are clearly enriched for the EDS (PAGE enrichment score  = 15) but so also, albeit to a lesser degree, are patients diagnosed with SSc (PAGE enrichment score  = 9.25). A possible explanation for this, once again, becomes clear upon the examination of gene expression sample by sample (Figure S1). In the Pendergrass data at least three patients scored as SSc have a high EDS score perhaps indicating, as within our own cohort, a further, as yet undetected, progression to the PH state. In datasets examining only PH (Bull et al. [11] and Rajkumar et al. [32] there is a clear and unambiguous enrichment (Table 3. Bull et al.; IPAH-7.7 and Rajkumar et al.; PAH-4.1, respectively) in their PH groups relative to controls. In the Rajkumar data, patients with idiopathic pulmonary fibrosis (IPF), a lung disease group not associated with PH did not display the EDS.

Interestingly, the platelet specific signature (Pl), while distinct in terms of gene content from the EDS (less than 5% gene overlap), was present in some patients with high EDS (Figure 3, Table S2), but did not show as much specificity for hypertension in our study or others (Figure S2) and for this reason was not pursued further. An additional data set was also evaluated which compared the gene expression between reticulocytes from cord blood and in the circulation [33]. The EDS was highly over-expressed in cord blood reticulocytes relative to peripheral blood supporting previous observations characterizing the presence of a less mature reticulocyte population in cord blood [22], [33] and supporting the identification of the EDS with reticulocyte development.

### Correlation of the EDS with Hemodynamic Measurements

**Figure 8:**
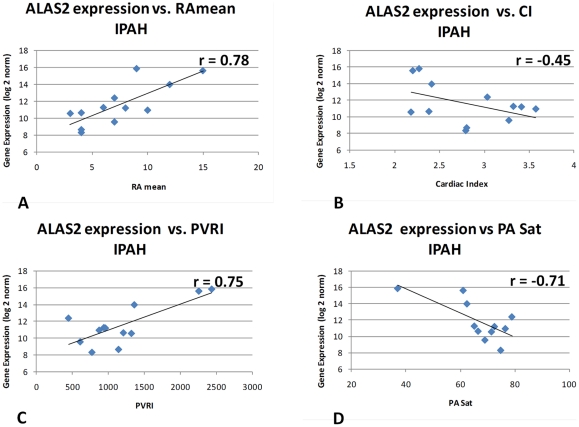
ALAS2 gene expression positively correlates with increasing disease severity in IPAH patients. EDS gene expression for 12 IPAH patients for whom hemodynamic measurements were available within four months of the date of blood draw. The clinical parameters of right atrial pressure (RAmean), cardiac index (CI), pulmonary vascular resistance index (PVRI), and pulmonary artery saturation (PA sat) were used. Disease severity in IPAH patients as indicated by increasing RAmean and PVRI are strongly correlated with increasing ALAS2 gene expression and negatively correlated with measures of healthy lung and coronary function such as PA saturation and Cardiac Index (CI).

It should be noted that despite the strong association of the EDS signature with the PH phenotype in our data and in others, only a subset of the patients in each group actually display elevated EDS levels. In order to better understand the association of EDS positive patients with disease progression, EDS gene expression regulation was examined for those patients for whom hemodynamic measurements were available within four months of the date of blood draw for the PBMC isolations used in this study (12 IPAH; 23 SSc-PAH). The clinical parameters of mean right atrial pressure (RAmean), cardiac index (CI), pulmonary vascular resistance index (PVRI), and pulmonary artery saturation (PA sat) were used. ALAS2 gene expression was taken as a primary biomarker representing the entire EDS (note the Pearson correlation r between ALAS2 and ERAF/AHSP gene expression, among the subjects used for this analysis, was 0.987, and 0.957 for IPAH and SSc-PAH, respectively), and was hence used for the purpose of computing correlations of the EDS with hemodynamic measurements (Figure 8). Disease severity in IPAH patients as indicated by increasing RAmean and PVRI were strongly correlated with increasing ALAS2 gene expression (r = 0.776, p = 0.003; r = 0.752, p = 0.0048, respectively). Conversely, measures of healthy lung and cardiac function such as PA saturation and Cardiac Index (CI) were negatively correlated with decreasing ALAS2 gene expression (r = −0.71, p = 0.0098; r = −0.449, p = 0.14, respectively). The consistency of these measurements suggests that there is indeed a direct correlation between disease severity and EDS gene expression in IPAH patients. Surprisingly, there were no corresponding significant correlations for any of these four hemodynamic parameters among the SSc-PAH patients (Figure S3) despite the clear and significant enrichment for EDS genes (see Figure 6A&B) in this group. The level of EDS gene over-expression among SSc-PAH patients was thus not seen to be directly related to the markers of PH disease status examined here and distinguishes IPAH and SSc-PAH for that reason.

## Discussion

Previous studies using PBMC have been used to identify PH-specific genes [11] as well as distinguishing between IPAH and SSc-PAH [12]. In general, however, these studies have shown considerable heterogeneity when examining directly the contrast in gene expression profiles in PBMC from SSc-PAH and SSc patients [13], [14]. For example, while Pendergrass et al. [13] focused on the increased expression of 9 genes that distinguished SSc-PAH from SSc, Risbano et al. [14] described 5 genes which when down-regulated distinguish SSc-PAH from SSc patients. In a comparison of the complete datasets from these studies in combination with results from this current study we found one gene, in particular, ALAS2, which is significantly over-expressed in SSc-PAH versus SSc in all three datasets. We present data here in support of the finding that one of the distinguishing molecular phenotypes in terms of gene expression between patients with PAH versus either healthy controls or patients with SSc alone, involves a distinctive signature uniquely associated with erythrocyte development. This signature was also present in patients with IPAH and SSc-PH-ILD in the current study and segregates with PH in multiple published clinical studies for which data is publicly available [11], [13], [14], [32]. We have demonstrated here that the EDS signature is quantitatively enriched and validated in at least four independently published datasets derived from microarray studies across multiple microarray platforms (Affymetrix, Illumina, and Agilent) in which the PBMCs from PH patients were tested.

In all cases (our data and others) only a subset of PH patients in the affected groups were EDS positive and in our data, at least, the presence of the EDS was associated with increasingly severe disease (as judged by hemodynamic parameters) for IPAH but not for SSc-PAH patients (Figure 8, Figure S3). The presence of the EDS signature induced by PAH may be indicative of increased red blood cell recruitment as part of a systemic response to severe chronic local hypoxia. An increase of up-regulated genes selectively expressed in erythrocytes/reticulocytes (including ALAS2 and ERAF/AHSP, and many other EDS genes) in whole blood was also noted to be consistent with previous observations of higher red blood cell counts (hematocrit) in obesity [34]. An increase in RBC trafficking may constitute a useful marker of PH disease, in general, and serve as a useful marker of increased disease severity specifically in IPAH patients. The lack of correlation of the EDS genes with hemodynamic measurements in the SSc-PAH patients is puzzling, particularly, as the signature as a whole is similarly enriched in patients from both disease groups. This perhaps reflects the different etiologies of these two different types of PH and emphasizes their distinct origins. Furthermore, we have previously reported that hemodynamic alterations are distinct between IPAH and SSc-PAH patients and do not always reflect, in the latter group, the severity of PAH [35], [36].

The cell specific source of the EDS remains an open question. In order to eliminate the likelihood that direct RBC contamination in the PBMC preparations used in this current study might account for the presence of the EDS, PBMC samples from both high and low EDS patients were subjected to multiple rounds of isotonic ammonium chloride hemolysis. This treatment lyses mature red blood cells with minimal effect on lymphocytes and does not appreciably affect nucleated red cells. In our test samples high EDS gene expression was not affected by this treatment (Figure S4).

High levels of gene expression for ALAS2 and ERAF are found almost exclusively in CD71+ erythroid progenitor cells, and the complete EDS signature (as defined here) appears to correlate especially well with genes known to be expressed in reticulocytes and particularly well in cell populations enriched for less mature reticulocytes (as in cord blood – Figure 5). Our hypothesis is that the EDS gene expression signature is derived from a population of nucleated reticulocytes which co-sediment with lymphocytes and monocytes in the PBMC fraction of Ficoll gradients.

The identity of the EDS corresponds closely to a gene expression signature reported by Ebert et al., [37] where the authors provide detailed gene expression information for an in vitro experiment in which bone marrow CD34+ cells were expanded under conditions which induced erythroid differentiation and major changes in gene expression were recorded pre- and post- induction. Our EDS gene list overlapped the induced erythroid gene list by over 50% including both the ALAS2 and ERAF genes (ALAS2, CA1, EPB42, ERAF, FECH, GLRX5, GSPT1, GYPB, GYPE, HBA2, MYL4, SELENBP1, SLC25A37, SNCA, TMCC2, and TSPAN5). Interestingly, a major difference between the Ebert induced erythroid signature and the PAH EDS is the very strong upregulation of IL8 recorded in the Ebert signature but not in the PAH-EDS (IL8 was upregulated sporadically and non-specifically across the entire PAH dataset). The reason for this discrepancy is not clear but worth noting given the otherwise very strong correlation between the two signatures.

Additional evidence for the source of the EDS in PBMCs has recently been obtained indirectly by others. Researchers from the Children's Hospital in Cincinnati reported the up-regulation of the expression of genes involved in the processes of hemoglobin synthesis and oxygen transport in the PBMCs of systemic juvenile idiopathic arthritis (sJIA) patients relative to healthy controls [38]. They suggested (as we do here) that this cluster of genes might represent the signature of immature nucleated RBCs that can copurify with PBMCs isolated on Ficoll gradients. The PH and the sJIA erythropoiesis signatures are highly overlapping (Figure S5) suggesting a common PBMC cell source. The Cincinnati team also mapped their experimentally derived EDS using bioinformatics methods to CD71+ immature erythroid precursor cells [39] and, in addition, they present data showing that patients with sJIA had significantly increased proportions of immature cell populations, including CD34+ cells, correlating highly with the strength of their measurements of the erythropoiesis signature. It has been independently demonstrated by *in vitro* expansion experiments that the potential, at least, for the production of large amounts of erythroid progenitor cells can be derived directly from PBMCs without additional purification [40], although whether the source of this expansion is derived from CD34+ cells remains uncertain [41]. Despite the strong indirect evidence presented by ourselves and others, the direct evidence for the expansion of CD71+ erythrocyte precursors in the periphery as the source of the EDS remains to be demonstrated.

The exact role of the EDS in specific disease states remains to be determined. Clearly there are strong associations between the EDS and PH and sJIA, but also the EDS has been observed in active lupus as well, and co-segregates with multiple additional immune-related signatures in a large asthma cohort study (unpublished data). In an ongoing longitudinal PAH study that we are currently conducting, it has become clear that the EDS as well as the immune response and platelet signatures are also variable in patients over time. Unfortunately, it is still too early for more than a speculative interpretation of these results. For now, it would appear that the EDS is an important new marker in chronic disease with the distinct property that in hypertension, at least, the expansion of immature precursor cells may actually constitute an active biological response to increasingly severe disease conditions.

## Materials and Methods

### Ethics Statement

All the samples used during this study were obtained following written informed consent from the donors. The Johns Hopkins University Institutional Review Board approved the conduct of this study.

### Study subjects

The cohort was comprised of subjects identified from the Johns Hopkins Pulmonary Hypertension Program and the Johns Hopkins Scleroderma Center as part of our center's Specialized Center for Clinically-Oriented Research program. Consecutive outpatients with a diagnosis of IPAH, SSc-PAH, SSc-PH-ILD and SSc were recruited and enrolled in the study between 2007 and 2010. Controls subjects were family and friends who accompanied subjects to clinic visits and had no known cardiovascular, pulmonary, or renal disease.

Limited or diffuse SSc was as previously defined [42]. PAH was defined as a mean pulmonary artery pressure (mPAP) of >25 mmHg, pulmonary artery wedge pressure (PAWP) ≤15 mmHg, and pulmonary vascular resistance (PVR) >3 Wood units [43], in the absence of other known causes of pulmonary hypertension. Results of pulmonary function tests (PFT) and high-resolution computed tomography (HRCT) of the chest closest to the date of the diagnostic RHC (right heart catheterization) were recorded. Percent of predicted results for all PFT data were calculated according to Crapo [44]. Patients with significant obstructive lung disease, defined as a forced expiratory volume in one second over forced vital capacity ratio (FEV_1_/FVC) <0.5 or 0.5–0.7 accompanied by radiographic evidence of emphysema, were excluded [45], [46]. Interstitial lung disease was defined by a combination of PFT and HRCT criteria as previously described in epidemiologic studies and clinical trials of PAH therapy [47], [48], [49]. Patients were classified as having SSc-PH-ILD if they met all the above criteria, and had a total lung capacity (TLC) of <60% of predicted or a TLC between 60% and 70% of predicted combined with moderate to severe fibrosis (grade 3–4) on HRCT [50]. The onset of scleroderma was defined by the first non-Raynaud's phenomenon manifestation.

Severity of PAH was assessed by routine functional and hemodynamic measurements obtained closest to the date of blood collection for genomic analyses. Subjects were then stratified by cardiac index dichotomized at 2.2 L/min/m^2^ based upon prior studies demonstrating poorer survival for PAH patients with a CI less than this value [51].

### Isolation of Peripheral Blood Mononuclear Cells

Venous blood was collected by simple venipuncture under aseptic conditions. All samples were processed within two hours of collection to minimize gene expression variations associated with longer sample incubation times [52]. PBMCs were separated by Ficoll density gradient, immediately lysed in Trizol reagent (Invitrogen, Carlsbad, California), and stored at −80°C.

### Purification of RNA

Total RNA was extracted using the Trizol Reagent method (Invitrogen, Carlsbad, California 92008, cat. no. 15596-026). Additional purification was performed on RNeasy columns (Qiagen, Valencia, CA 913555, cat. no. 74104). The quality of total RNA samples was assessed using an Agilent 2100 Bioanalyzer (Agilent Technologies, Palo Alto, CA).

### Microarray Analysis

RNA samples were labeled according to the chip manufacturer's recommended protocols. In brief, for Illumina, 0.5 µg of total RNA from each sample was labeled by using the Illumina TotalPrep RNA Amplification Kit (Ambion, Austin, TX 78744-1832, cat. no. IL1791) in a two step process of cDNA synthesis followed by *in vitro* RNA transcription. Single stranded RNA (cRNA) was generated and labeled by incorporating biotin-16-UTP. 0.75 ugs of biotin-labeled cRNA was hybridized (16 hours) to Illumina Sentrix Human HT12_v3 BeadChips (Illumina, San Diego, CA 92121-1975, cat.no. BD-103-0203). The hybridized biotinylated cRNA was detected with streptavidin-Cy3 and quantitated using Illumina's BeadStation 500GX Genetic Analysis Systems scanner**.** The expression data discussed in this publication have been deposited in NCBI's Gene Expression Omnibus [53] and are accessible through GEO Series accession number GSE 33463 (http://www.ncbi.nlm.nih.gov/geo/query/acc.cgi?acc=GSE 33463).

### Quantitative RT-PCR (QRT-PCR) Analysis

cDNA was obtained from total RNA using the cDNA Archive Kit according to the manufacturer's protocol (Applied Biosystems, Foster City, CA). Probes and primers were designed and synthesized by Applied Biosystems. All PCR amplifications were carried out in triplicate on an ABI Prism® 7300 Sequence Detection System, using a fluorogenic 5′ nuclease assay (TaqMan® probes). Relative gene expressions were calculated by using the 2^−ΔΔCt^ method as described in [54]. The ΔCt value of each sample was calculated using 3 endogenous control genes (GAPDH, ACTB, and PGK1).

### Analytical Methods and Statistical Analysis

A single intensity (expression) value for each Illumina probe on the array was obtained using Illumina BeadStudio software with standard settings and no background correction. The expression values for all the probes for each sample were scaled to have median 256 (2^8^) and then log (base 2) transformed before performing statistical analysis. Analysis for differential expression between pairs of disease groups and between individual disease groups and the group of controls was carried out for each Illumina microarray probe.

Illumina probes (and consequently, the corresponding genes) considered to be significantly differentially expressed between two groups of samples were those satisfying the three criteria: (i) Two-sided Welch t-test p-values less than or equal to 0.01 [55]; (ii) a Benjamini-Hochberg false discovery rate (FDR) less than or equal to 0.1 [56]; and (iii) a fold change above 1.5 or below 1/1.5 (calculated using geometric means). Statistical testing for differences in expression levels of a given probe between two groups was not done unless at least 80% of the samples in the group with the higher average expression level for that probe had Illumina detection p-values <0.01 (thus avoiding false positives based on background noise and also reducing the number of statistical tests for the subsequent false discovery rate calculation). A comprehensive spreadsheet of group comparison results is given in Table S1.

Heat maps (and the ordering by hierarchical clustering of the samples and the genes in heat maps) were based on normalization of the expression values for each sample using z transformation [55], [57], [58] (utilizing only the probes which had an Illumina detection p-value <0.01 for least one sample, and for which there was an available gene symbol), followed by z-transformation of the normalized expression values for each Illumina probe across all samples. Hierarchical clustering was performed using the Cluster and TreeView software programs [59]. The clustering algorithm was set to complete linkage clustering using uncentered correlation.

Gene set analysis was carried out using the web-PAGE tool [16] (http://dpwebpage.nia.nih.gov/PAGE/index.html) with the p-value cutoff set to 0.01 and the minimum number of genes per gene set equal to 10. The input used was either a z ratio (z transformation of the difference between the means computed for two groups [60] (Table 2, Table 3-JHU data, Figure 7) for all internal data; or a simple difference metric was computed and used for all external data (Table 2). One exception was the use of all row normalized data on a sample by sample basis to generate the functional genomic landscape as illustrated in Figure 4A. In this variation of the gene set algorithm, gene sets are tested against the complete expression data for each sample, enrichment scores map the relative abundance levels of groups of genes which can then be compared for variation between samples and groups of samples as seen. The gene sets used are as described in the corresponding Table and Figure legends.

Evaluation of lists of differentially expressed genes for enrichment in predefined categories and functional groups of genes was carried out using the NIH functional analysis tool DAVID [61], and the Broad Institute Molecular Signatures Database MSigDB facility [62].

### Associating hemodynamic measurements with gene expression

Correlation between expression levels of a given gene and the values of a selected clinical variable for a specified group of n subjects was evaluated as the Pearson product-moment correlation coefficient r estimating the population correlation coefficient ρ. Tests of the null hypothesis ρ = 0 were conducted using the customary t-test depending on r and n.

## Supporting Information

Table S1 **PAH significance tests, gender specific EDS stats, siglists-unique, UR functional enrichment, DR functional enrichment.**
(XLSX)

Table S2 **EDS and Platelet specific genes.**
(XLSX)

Table S3 **Demographic and clinical data for subjects.**
(XLSX)

Figure S1 **EDS genes in published PH gene expression datasets.**
(TIF)

Figure S2 **Platelet signature in published PH gene expression datasets.**
(TIF)

Figure S3 **Correlations of ALAS2 gene expression with clinical measurements in SSc-PAH patients.**
(TIF)

Figure S4 **RBC lysis treatment shows no effect on EDS gene expression.**
(TIF)

Figure S5 **EDS genes over-expressed in sJIA dataset.**
(TIF)
