# Supplementary figures and images for: Erythroid-Specific Transcriptional Changes in PBMCs from Pulmonary Hypertension Patients
Source: PLoS One. 2012 Apr 24;7(4):e34951. doi: 10.1371/journal.pone.0034951 (PMC3335832; doi:10.1371/journal.pone.0034951)

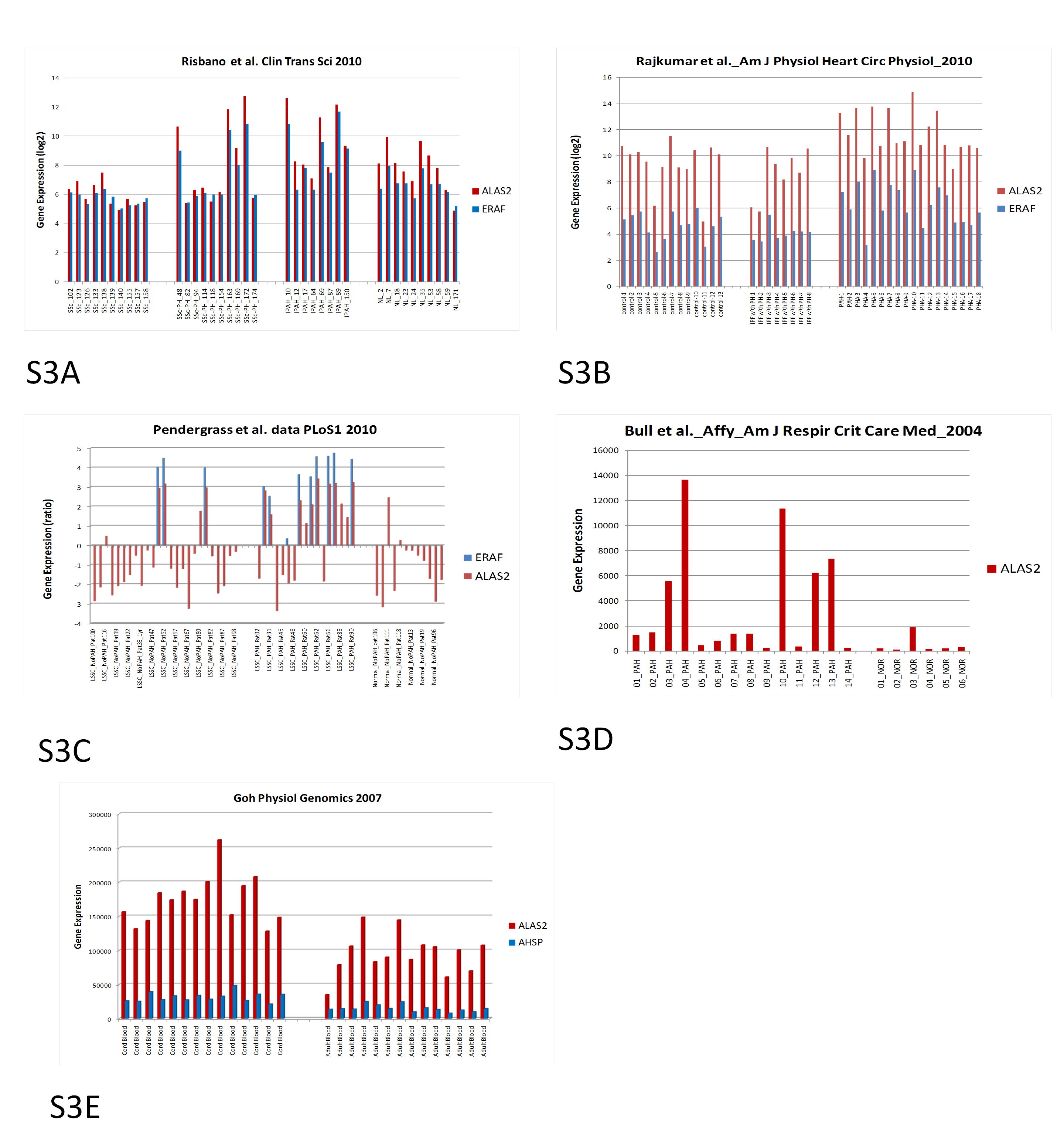

Supplement: Figure S1 — EDS genes in published PH gene expression datasets. (TIF) [file pone.0034951.s004.tif]

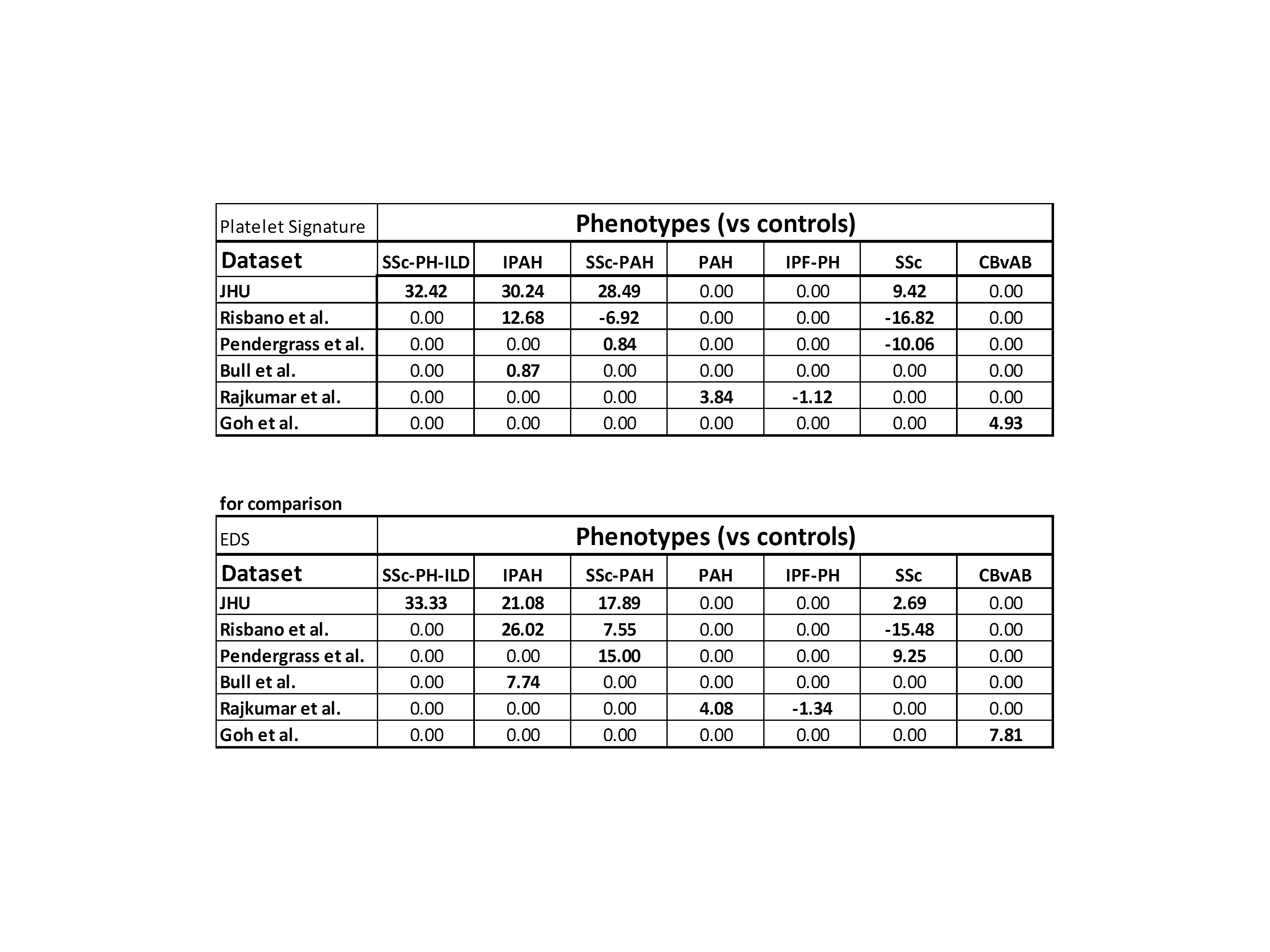

Supplement: Figure S2 — Platelet signature in published PH gene expression datasets. (TIF) [file pone.0034951.s005.tif]

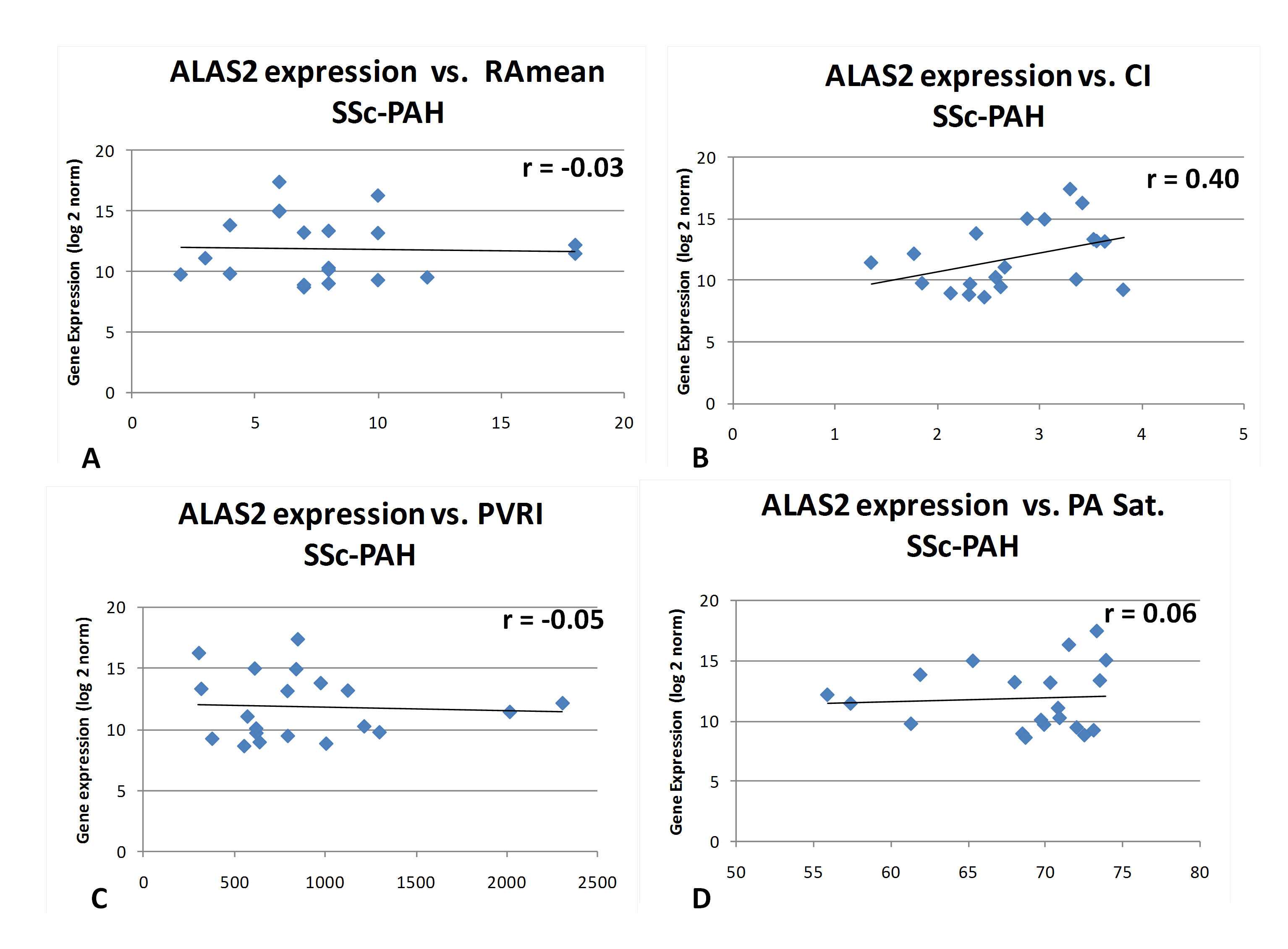

Supplement: Figure S3 — Correlations of ALAS2 gene expression with clinical measurements in SSc-PAH patients. (TIF) [file pone.0034951.s006.tif]

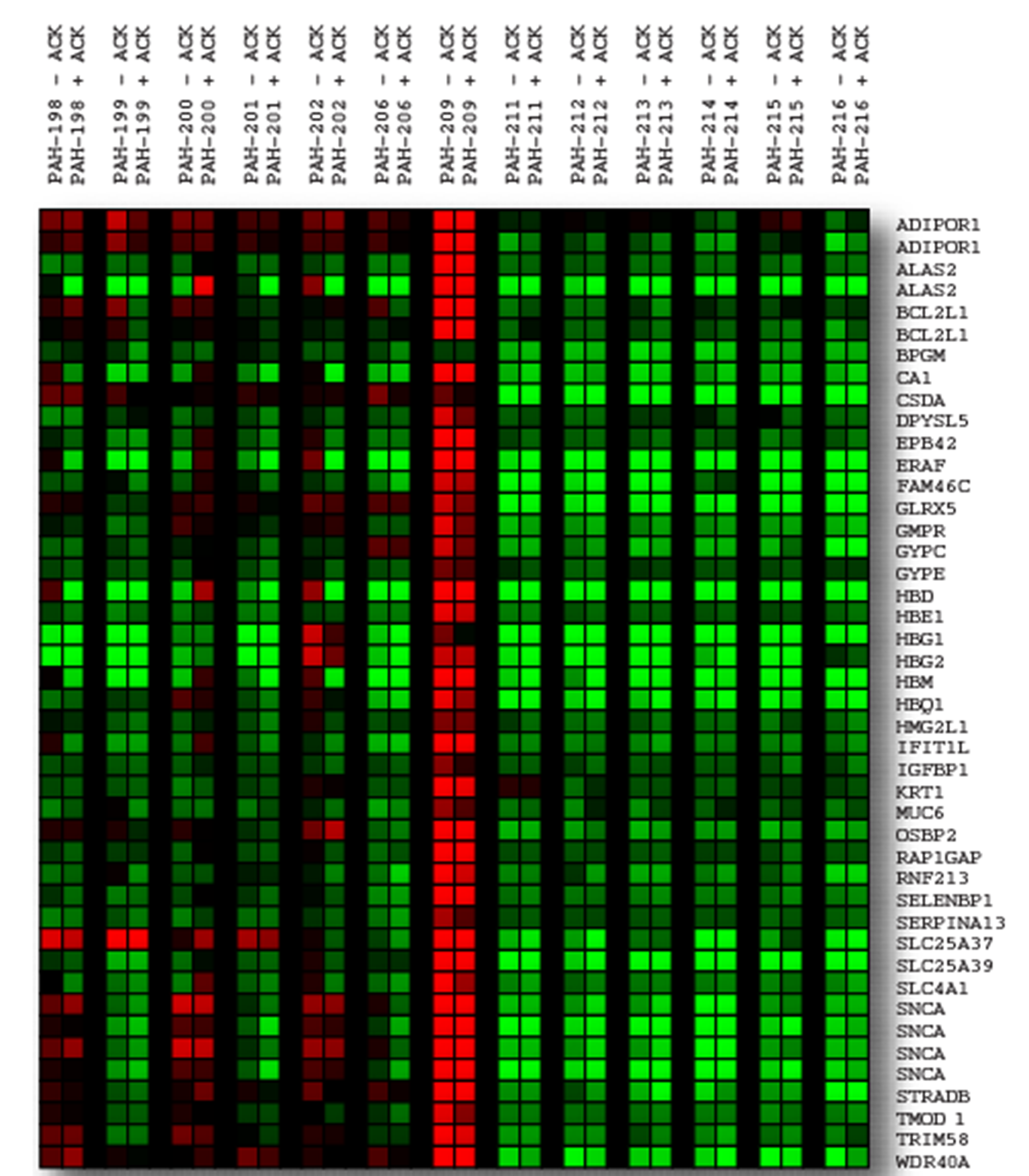

Supplement: Figure S4 — RBC lysis treatment shows no effect on EDS gene expression. (TIF) [file pone.0034951.s007.tif]

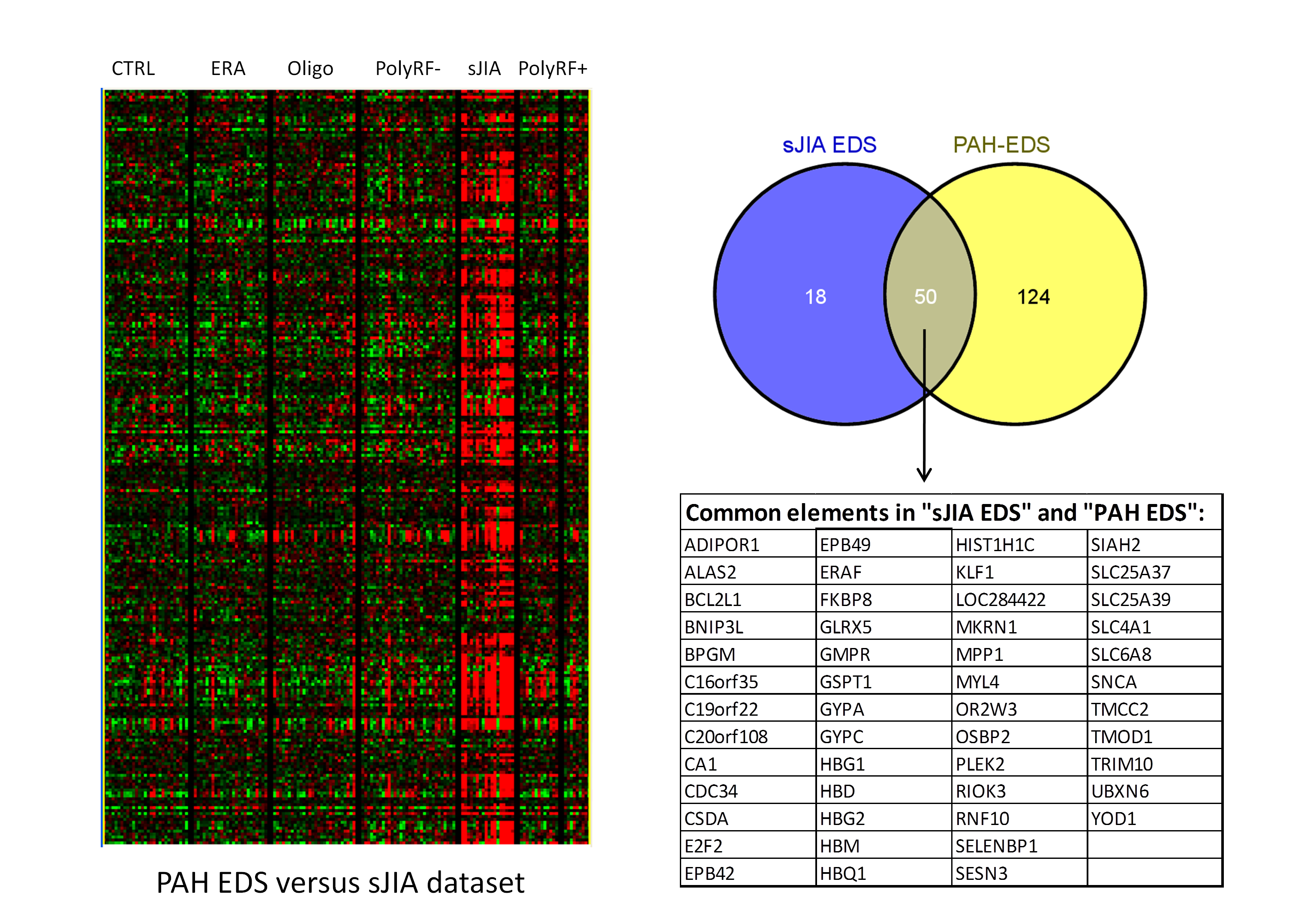

Supplement: Figure S5 — EDS genes over-expressed in sJIA dataset. (TIF) [file pone.0034951.s008.tif]
